# A Strong Policy Outlined

**Published:** 1920-11-27

**Authors:** 


### THE FUTURE OF MEDICAL RESEARCH.

### A Strong Policy Outlined.

The address to the Fellows of the Royal Society of Medicine, recently given by Sir Almroth Wright, was characteristically delivered, cordially received, and, as already explained, represented the occasion of the first presentation of the Society's newly instituted gold medal. But the importance of the meeting appears to lie as much in the singularly apt review which the speaker made as in the ceremony itself. There has been so much lay misunderstanding upon the question of what medical research really is, and at the same time such urgent appeals that they should support it financially, that certain remarks by Sir Almroth Wright are well worth careful study.

In discussing the types of thought prevalent to-day he points out that every man and every woman feels competent to make a generalisation; the trouble is that so very few people can make big generalisations. The man who is most likely to do this is he who makes very few mistakes as a diagnostician. Having made a generalisation, the next step is to verify it. Four out of every five generalisations believed by a man are wrong, and nine out of ten believed by women are wrong. The scientific man is the man with a scientific imagination who afterwards knows how to verify. The object of medical research is to get generalisations if possible, but one cannot command them. Verification of the experiment, however, can be commanded. The man who makes a genetic suggestion does not advance things unless he devises a means of testing it and has the skill to carry it out, always with the object of improving prophylaxis and treatment. Experiment is the intellectual machinery by which this is done. Most of the experiments which are done are futile; they do not lead to results. If a layman—a quack, for instance—wants to experiment as to whether a particular thing does good, he gets together a number of cases which he regards as all alike, his first fallacy. If he desires to ascertain whether violet leaves are good for cancer he gets together cases, a number of which probably are not cancer at all. And the experiment is invalidated because it is purely at random, undertaken without reason. A layman has no power, either, of judging as to the efficacy of a drug.

Even in the case of Carrel's treatment of wounds every one had a chance of testing it at the Front, but it had many factors in it, all of which were not duly recognised. The judgment of the medical man not trained in laboratory methods is not always safe.

Something of the same limitations must inevitably arise in connection with statistical work, but it is unnecessary to lead the reader into the details. From all points of view Sir Almroth Wright summarises the fact that laboratory work as such must stand as the most vital section of a research unit, and proceeds therefrom to build up his picture of the ideal. No single clinical section can stand without the assistance of the others, and although surgical, medical, gynæcological, and other clinical "units" have been instituted in many quarters, yet the lack of this type of development is most notable in connection with the laboratory service of the hospitals, a medical factor of the greatest importance.

Accounts reflect upon the relative isolation or congestion of many existing so-called pathological "sides," and the relatively junior rank accorded to their work as well, show the foundation for the demand that, instead of finding in every hospital only a clinical staff which is available for treatment, there must also be a laboratory research staff, whose function would be to test new things, to make generalisations, and so extend knowledge by subsequent verification. That condition of things does not exist anywhere at present save at St. Mary's.

How should a Research Service be Organised?

Laboratories detached from hospitals do not turn out good work, as they suffer from paucity of ideas without the continual stimulus of hospital material.

The only person who can direct men to do useful research is a person who knows the methods from having himself been engaged in laboratory work. A surgeon or physician who has not had such training has no place on the directing staff; the latter must consist entirely of men brought up to the trade of laboratory investigation, and the higher workers in this should have their share in directing the policy. At present the Medical Research Council consists of those who are not whole-time laboratory employees; every whole-time laboratory worker is excluded. Certainly the president of a research organisation should not be a busy politician or any other layman, but a person occupied with and eminent in science.

